# Genomic characterization of novel orthohepeviruses in shrews and rats from Kenya

**DOI:** 10.1099/mgen.0.001538

**Published:** 2025-12-05

**Authors:** Carol Vannesa Nawenja, Griphin Ochieng Ochola, Vincent Obanda, Sheila Ommeh, Xing-Lou Yang, Yan Zhu, Bei Li, Jie-Wen Deng, Bernard Agwanda, Ben Hu

**Affiliations:** 1 State Key Laboratory of Virology and Biosafety, Wuhan Institute of Virology, Chinese Academy of Sciences, Wuhan, PR China; 2 University of Chinese Academy of Sciences, Beijing, PR China; 3 Department of Mammalogy, National Museums of Kenya, Nairobi, Kenya; 4 Veterinary Science and Laboratories Department, Wildlife Research and Training Institute, Naivasha, Kenya; 5 Center for Animal Science, Queensland Alliance for Agriculture & Food Innovation, the University of Queensland, Brisbane, Queensland, Australia; 6 Key Laboratory of Genetic Evolution & Animal Models, Kunming Institute of Zoology, Chinese Academy of Sciences, Kunming, PR China

**Keywords:** cross-species transmission, Kenya, orthohepevirus, rat, shrew

## Abstract

Rodents and shrews are two groups of small mammals living in proximity with humans and have been known to harbour a variety of zoonotic pathogens. Cross-species transmission of hepeviruses from animals, particularly the recent sporadic emergence of human infections by rat-borne hepeviruses, has posed a growing threat to human health. Here, we report the full-genome identification of two orthohepeviruses in African giant shrew (*Crocidura olivieri*) and black rat (*Rattus rattus*) from Kenya, named Co-KY2016 and Rr-KY2016, respectively, the partial polymerase gene sequences of which were previously described. Co-KY2016 is highly distinct from representative strains of all currently recognized orthohepevirus species, sharing less than 55% overall genome identity and possibly representing at least a novel virus species together with other recently reported shrew hepeviruses. Rr-KY2016 shared higher similarity with other hepeviruses of rat origin in the *Rocahepevirus* genus, including human-infecting strains. Our results provide more evidence that rats and shrews are reservoir hosts of hepeviruses and support previous findings that different hepeviruses have undergone co-speciation with their hosts during evolution. This study increases our understanding of the distribution and genetic diversity of hepeviruses in wildlife as well as their spillover risk in Africa. It also highlights the importance of identifying hepeviruses in rodents, shrews or other wildlife and investigating possible zoonotic transmission of hepeviruses to mitigate the emergence of future diseases that could threaten public health.

**Impact Statement**

This study provides the first report of the complete genomes of rodent and shrew hepeviruses in Kenya. Although different groups have previously documented newly identified shrew hepeviruses in recent years, our study proposes for the first time that these shrew hepeviruses, including the one we discovered in Kenya, may belong to a novel species or even a new orthohepevirus genus. Meanwhile, the Kenyan rat hepevirus that we found shows close similarity to another rocahepevirus strain which caused human hepatitis. Our findings highlight the importance of rodents and shrews as natural reservoir hosts of hepeviruses and the potential risk of hepevirus transmission from wildlife in Africa. The study provides reference for prevention and precaution of future emerging infectious diseases associated with novel zoonotic hepeviruses. It also underscores the value of proactive viral discovery and targeted surveillance among those wildlife reservoirs.

## Data Summary

Full-length genome sequence data of the two shrew and rat orthohepeviruses that support the findings of this study have been deposited in GenBank with accession numbers PV861666 and PV861667.

## Introduction

Hepeviruses refer to a group of quasi-enveloped, single-stranded and positive-sense RNA viruses belonging to the *Hepeviridae* family, including the classic human hepatitis E virus (HEV) and different HEV variants from other animals. These hepatotropic viruses can infect a wide variety of mammalian and avian species and cause acute viral hepatitis in some hosts, including humans [1,2]. HEV annually infects about 20 million human cases worldwide and leads to over 40,000 deaths, posing serious global health threats, especially in low- and middle-income countries with poor sanitation and hygiene conditions where faecal–oral route has been suggested as the main route of transmission [3,4]. Although acute hepatitis E is generally self-limiting in humans, cases of HEV infection with severe symptoms have been reported in different groups of immunocompromised individuals, and chronic HEV infection has become a significant public health problem with increasing concern [5,6].

Members of the *Hepeviridae* family are highly diverse, with all mammalian and avian hepeviruses belonging to the *Orthohepevirinae* subfamily. According to the taxonomy of International Commitee on Taxonomy of Viruses (ICTV), orthohepeviruses consist of four genera, namely *Paslahepevirus*, *Rocahepevirus*, *Avihepevirus* and *Chirohepevirus*, the latter two of which are mainly found in birds and bats, respectively [7]. As these viruses have a broad host spectrum, zoonotic transmission is an important source of hepevirus infection in humans. So far, zoonotic infection of hepeviruses has been primarily associated with the genus *Paslahepevirus*. Of the eight genotypes within the species *Paslahepevirus balayani*, genotypes 1 and 2, known as the classic human HEV, are the prototypical causative agents of human hepatitis E and are mainly restricted to the human host, while other genotypes have non-human reservoirs [8,9]. Among them, genotypes 3 and 4 (termed swine HEV) are mainly carried by pigs and wild boars and are responsible for the majority of zoonotic HEV cases, which have been associated with consumption of either raw or undercooked meat [10]. However, more recently, sporadic human infections caused by HEV variants from rats (termed rat HEV) that belong to the genus *Rocahepevirus* (previously known as HEV-C) have been independently documented worldwide without a clear transmission route, raising new public health concerns [11]. Meanwhile, a growing number of novel HEV variants have been discovered, and more hepevirus reservoir hosts have been identified, including moose, rabbit, tree shrew and shrews (termed moose HEV, shrew HEV, etc.) [12]. The recognition of the extended host range of hepeviruses and the recent frequent occurrence of spillover from rodents necessitate the need for continuous surveillance and identification of novel zoonotic hepeviruses in wildlife.

We previously detected 250 bp partial RNA-dependent RNA polymerase sequences of two novel orthohepeviruses in shrew and rat samples, respectively [13]. Here, we report on full-length genome characterization and phylogenetic analysis of these two viruses. Our findings provide new information for understanding the genetic diversity and evolution of *Hepeviridae*, as well as for the precaution of zoonotic HEV emergence in Africa.

## Methods

### Sample collection

Fieldwork was carried out in Kenya during 2016. Rodents and shrews were baited and captured using Sherman live traps. Handling of the captured animals was according to the guidelines by the American Society for Mammalogists and the National Museums of Kenya [14]. Upon retrieval from traps, animals were euthanized, dissected and internal organs were collected in 2-ml collection tubes, which were later temporarily stored in liquid nitrogen for transportation and later in −80 °C for permanent storage.

### Sample processing and RNA extraction

Tissue samples were lysed in Hanks’ Balanced Salt Solution using the QIAGEN TissueLyser II as per the manufacturer’s instructions (QIAGEN, Germany). Lysed samples were briefly spun in a 4 °C pre-cooled centrifuge, and supernatant was used for RNA extraction using High Pure Viral RNA Kit (Roche, Germany). RNA was stored at −80 °C or subsequently used in downstream experiments.

### Whole viral genome sequencing

RNA of two shrew and rat liver samples positive for hepevirus, as identified in previous screening experiments [13], was used in preparation of sequencing libraries, which were then subjected to next-generation sequencing (NGS) performed on a HiSeq 3000 sequencer (Illumina). The paired-end sequencing was conducted with a read length of 150 bp. Raw sequencing data were processed using fastp (v0.23.4) to remove adaptor sequences and low-quality bases. Quality-controlled reads were aligned to the silva database using Bowtie2 (v2.5.2) to filter out rRNA. The remaining clean reads were assembled into contigs using megahit (v1.2.9), with default parameters and the minimum contig length set to 1,000 bp. Resulting contigs were aligned to the National Center for Biotechnology Information (NCBI) nucleotide database using blastn for identification. To fill gaps in the genome, we employed nested PCR using Platinum Taq DNA Polymerase Kit (Invitrogen, Carlsbad, USA) with primers designed based on known genomic regions. The primer sequences are available in Table S2, available in the online Supplementary Material. PCR thermocycling conditions were set at 94 °C for 2 min followed by 40 cycles of 94 °C for 20 s, 55 °C for 30 s and 72 °C for 30 s. A final extension step was set at 72 °C for 5 min. The annealing temperature was set at 5 °C below the melting temperature of the primer pair used. The two rounds of PCR followed a similar approach, with the PCR product from round one used as a template in round two. PCR products were run on 2% agarose gel and positive samples were sent for Sanger sequencing (Sangon Biotech). 5′ and 3′ ends of the genomes were determined using the HiScript-TS 5′/3′ RACE Kit (Vazyme, China) together with Prime Star High Script Kit (Takara) following the manufacturer’s instructions.

### Genomic sequence analysis and phylogenetic analysis

The complete genome sequences were analysed and annotated using BioEdit v.7.2 (Informer Technologies, USA). ORF prediction software tool (NCBI) was used to predict the ORFs. The newly identified orthohepevirus genome sequences were compared and aligned with representative orthohepevirus sequences downloaded from GenBank, and sequence identity was calculated at nt and aa levels. A genome sequence identity plot was created in SimPlot v.3.5.1. Phylogenetic analyses with ORF1 and ORF2 proteins were performed using mega 7 software with 1,000 bootstrap replicates.

## Results

### Host species, sampling location and naming of the novel viruses

We previously detected partial RdRp sequences of two hepeviruses in liver samples from an African giant shrew (*Crocidura olivieri*) and a black rat (*Rattus rattus*) collected in Kitale town, western Kenya [13]. Subsequently, in this study, we conducted NGS and obtained full-length genome sequences of these two distinct hepeviruses by *de novo* assembly, targeted PCR and 5′/3′-RACE. We tentatively rename these two viruses as shrew HEV Co-KY2016 and rat HEV Rr-KY2016, based on the abbreviation of host species, geographic origin and sampling date.

### Genome organization and sequence comparison

**Fig. 1.**
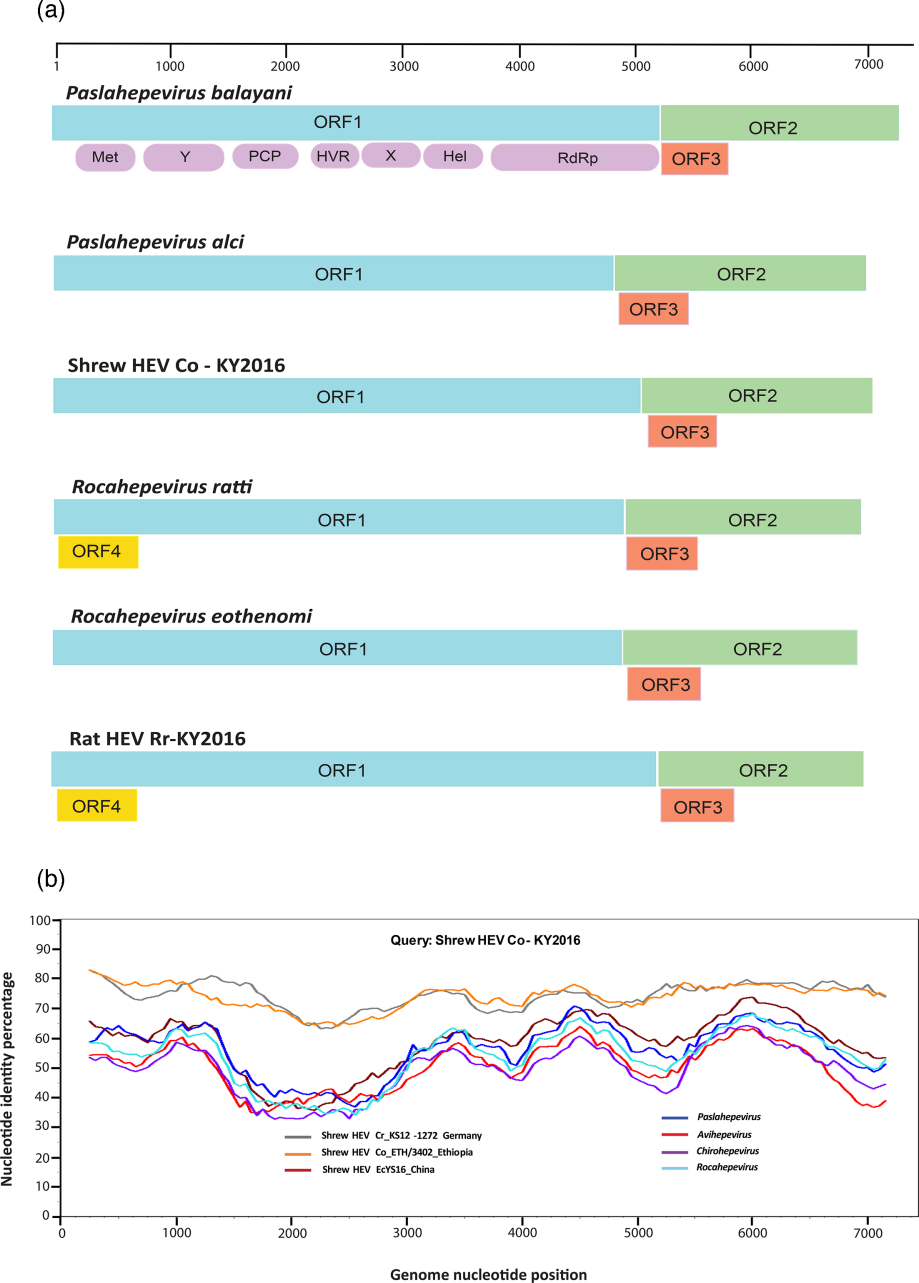
Comparative genome sequence analyses of the novel shrew and rat HEVs. (a) Comparison of genome organization of shrew HEV Co-KY2016, rat HEV Rr-KY2016 and representative members within the family *Hepeviridae*. The name of the virus or virus species is shown at the top of each genome. Colour-coding for three typical hepeviral ORFs as follows. Blue, ORF1; green, ORF2; orange, ORF3. The putative ORF4 of *Rocahepevirus ratti* is depicted in yellow. Genome scale is indicated in bases at the top. (b) Genome sequence identity plot of shrew HEV Co-KY2016 and selective orthohepevirus strains. An identity plot was created in SimPlot v.3.5.1 with a window size of 500 bp and a step size of 50 bp. GenBank accession numbers of other shrew HEV strains and representative strains of different hepevirus genera are as follows: OR713884 (shrew HEV KS12-1272), PQ489619 (shrew HEV ETH/3402), PP533472 (shrew HEV EcYS16), M73218 (*Paslahepevirus*), AY535004 (*Avihepevirus*), JQ001749 (*Chirohepevirus*) and GU345042 (*Rocahepevirus*).

The full-length genome of shrew HEV Co-KY2016 comprises 7,019 nt excluding the poly A tail, with its genome ends harbouring the 5′ and 3′ UTRs. The genome is divided into three major ORFs predicted as ORF1, ORF2 and ORF3 that encode the nonstructural protein, capsid protein and phosphoprotein, respectively (Fig. 1a). The genome sequence identity between shrew HEV Co-KY2016 and previously reported hepeviruses is less than 75%. It shows the highest similarity to the other two recently discovered shrew HEVs, namely KS12-1272 from *Crocidura russula* in Germany and ETH/3402 from *C. olivieri* in Ethiopia, sharing 74.5% and 74.2% overall genome identity, respectively (Table 1 and Fig. 1b). The ORF1 and ORF2 proteins of Co-KY2016 shared 85.4–87.1% and 88.6–89.4% sequence identities with the above two shrew HEVs, compared to 73.3–73.5% and 76.4–77.0% sequence identities at nucleotide level (Table 1). Co-KY2016 is more varied from another shrew HEV named EcYS16, which was more recently identified in *Episoriculus* shrew from China. They share only 58% overall genome identity, while their ORF1 and ORF2 gene sequence identities are around only 60%. Furthermore, the Kenyan shrew HEV is highly distinct from other known hepeviruses. The overall sequence identities of Co-KY2016 and representative viruses in the four genera of *Orthohepevirinae* are all below 55% (Table 1 and Fig. 1b).

**Table 1.** Comparison of the full-length genome, ORF1 and ORF2 sequence identity percentage between the novel shrew HEV Co-KY2016 detected in Kenya and representative virus strains within the *Orthohepevirinae* subfamily from various host species, including other HEV variants recently reported from shrews

Strain name	GenBank accession no.	Host species	Virus species	Sampling location	Sequence identity (%)				
					Full-length genome	ORF1		ORF2	
						Nucleotide	Amino acid	Nucleotide	Amino acid
KS12-1272	OR713884	*Crocidura russula*	Unassigned species	Germany	74.5	73.5	87.1	77.0	88.6
ETH/3402	PQ489619	*Crocidura olivieri*	Unassigned species	Ethiopia	74.2	73.3	85.4	76.4	89.4
EcYS16	PP533472	*Episoriculus caudatus*	Unassigned species	China	58.1	56.4	57.8	62.2	61.6
Yunnan-2013	KR905549	*Tupaia belangeri*	Unassigned species	China	53.5	52.5	48.6	58.1	58.0
AlgSwe2012	KF951328	*Alces alces*	*Paslahepevirus alci*	Sweden	53.8	52.9	48.5	56.4	57.2
Burma	M73218	*Homo sapiens*	*Paslahepevirus balayani* (genotype 1a)	Burma	54.2	53.4	48.7	56.6	57.2
Meng	AF082843	*Sus scrofa*	*Paslahepevirus balayani* (genotype 3a)	USA	53.5	51.9	48.7	57.2	58.0
DcHEV/178C	KJ496143	*Camelus dromedarius*	*Paslahepevirus balayani* (genotype 7a)	UAE	53.7	52.8	49.1	55.9	57.6
rat/R63/DEU/2009	GU345042	*Rattus norvegicus*	*Rocahepevirus ratti*	Germany	53.6	52.1	48.2	56.7	55.3
Em40/LuXi/2014	MG020024	*Eothenomys melanogaster*	*Rocahepevirus eothenomi*	China	53.4	52.4	48.5	55.8	53.7
little egret/kocsag02/2014/HUN	KX589065	*Egretta garzetta*	*Avihepevirus egretti*	Hungary	49.1	49.5	43.4	51.1	49.1
Avian_HEV	AY535004	*Gallus gallus*	*Avihepevirus magniiecur*	USA	49.2	48.9	44.1	51.8	48.7
DesRot/Peru/API17_F_DrHEV	MW249011	*Desmodus rotundus*	*Chirohepevirus desmodi*	Peru	49.2	48.2	41.3	53.0	50.5
BatHEV/BS7/GE/2009	JQ001749	*Eptesicus serotinus*	*Chirohepevirus eptesici*	Germany	48.0	47.2	40.6	52.5	50.6
Bat Rf-HEV/Shanxi2013	KJ562187	*Rhinolophus ferrumequinum*	*Chirohepevirus rhinolophi*	China	49.9	49.1	42.3	52.7	50.8

Compared with the shrew HEV, rat HEV Rr-KY2016 demonstrates relatively high homology with known hepeviruses. It exhibits above 80% full-length genome sequence identity to a number of previously described *Rocahepevirus* strains, which were reported in rats or humans (Table S1). Strikingly, it shared the highest overall sequence identity (86.9%) with a rocahepevirus infecting humans in France. It is very similar in size and genomic organization to other rat HEVs. In addition to ORF1, ORF2 and ORF3, it has an additional ORF4 which overlaps ORF1 at the N terminus, albeit with a late start codon. This additional ORF is conserved among most viruses of the species *Rocahepevirus ratti* (Fig. 1a). However, the function of ORF4 remains unclear [15]. The sequence identities between Rr-KY2016 and selected genotype C1 strains of *Rocahepevirus ratti* range from 76.7 to 85.9% and 79.7 to 89.2% at the nucleotide level for ORF1 and ORF2, respectively, while the amino acid identities increase to 88.0–95.0% and 92.4–96.4%. In contrast, its similarity to C2 and C3 genotypes and to other *Rocahepevirus* species is significantly lower. These results classified Rr-KY2016 as a novel variant within the species *Rocahepevirus ratti*.

### Phylogenetic analysis and taxonomic placement

**Fig. 2.**
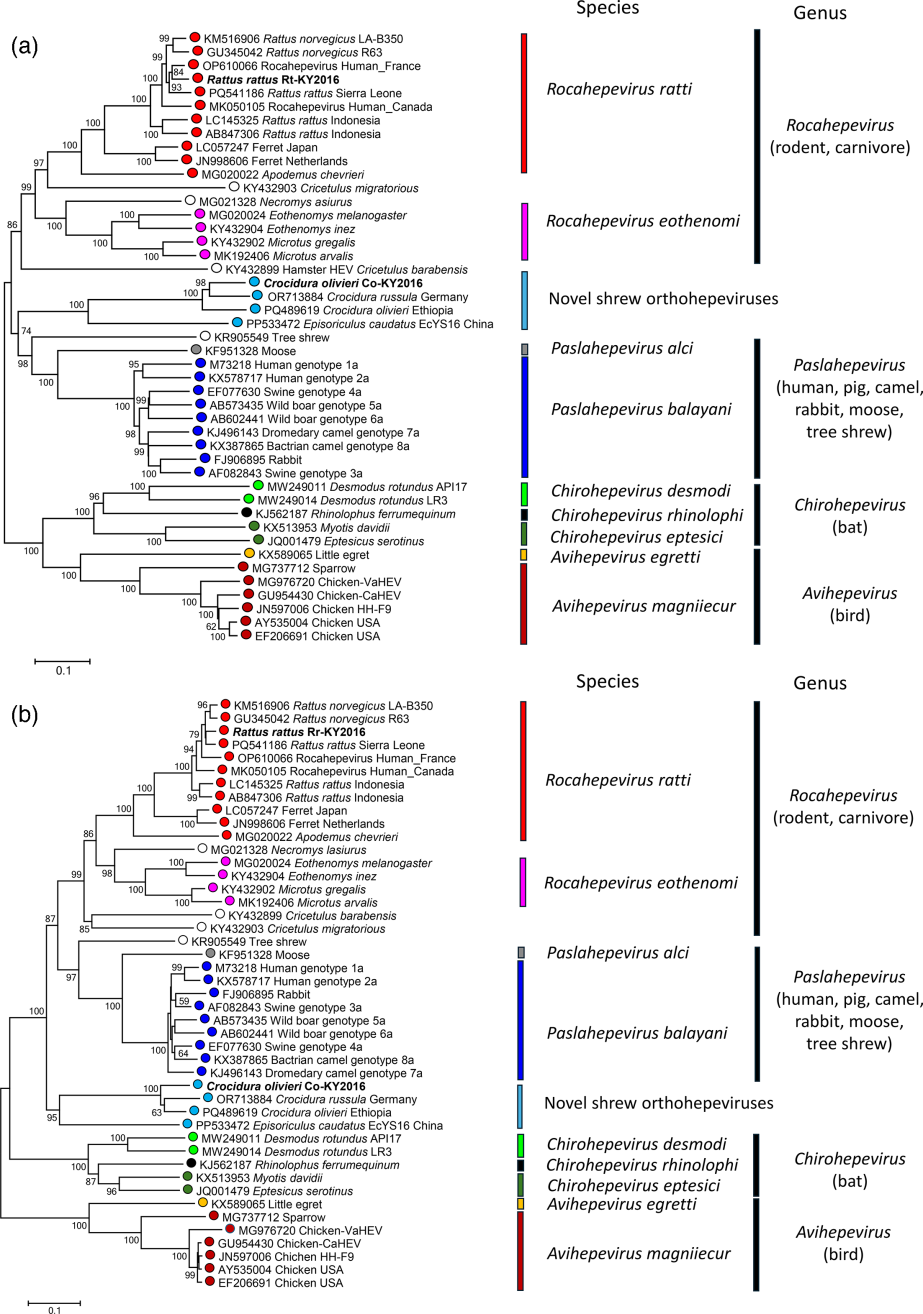
Phylogenetic trees of the ORF1 and ORF2 sequences of the novel shrew HEV, rat HEV and related orthohepeviruses. (a) Neighbour-joining tree of the full-length ORF1 protein sequences. (b) Neighbour-joining tree of the full-length ORF2 protein sequences. Trees were constructed using a Poisson model with pairwise deletion, based on amino acid sequences of ORF1 and ORF2 of shrew HEV Co-KY2016, rat HEV Rr-KY2016 and 43 representative members within the family *Hepeviridae*. Viruses belonging to different species assigned by ICTV are represented by circles in different colours. The host species information of all listed hepeviruses from rodents and shrews is shown. The newly sequenced shrew and rat HEVs are highlighted in bold. Scale bars, amino acid substitutions per site.

We further investigated the phylogenetic relationship among the newly sequenced shrew and rat HEVs and previously known orthohepeviruses using their ORF1 and ORF2 sequences. The *Rocahepevirus ratti* species contains HEV variants from both rodents and carnivores. Strains of *Rocahepevirus ratti* can be divided into three branches, represented by three genotypes of this species, which are found in rats (*Rattus* spp.), ferrets and field mice (*Apodemus* spp.) and were formerly designated as HEV-C1, HEV-C2 and HEV-C3, respectively [16]. In both ORF1 and ORF2 trees, the rat HEV Rr-KY2016 falls into the HEV-C1 genotype. It is clustered with other HEV-C1 strains identified in *Rattus rattus* and *Rattus norvegicus* from different continents, as well as some strains causing human infections but probably originating in rats. It shows the closest phylogenetic relationship to the human-infecting HEV-C1 strain reported in France and another *Rattus rattus* hepevirus from Sierra Leone in western Africa (Fig. 2). The phylogenetic analysis revealed that four shrew HEVs identified by independent teams, including Co-KY2016, formed a novel clade of orthohepeviruses. Within this clade, the Kenyan shrew HEV is more closely related to the other two shrew HEV strains also from *Crocidura*, but with a different geographical origin. One was discovered in Ethiopia, while the other was from Germany. These three *Crocidura* HEVs form a sub-clade separate from the HEV variants found in *Episoriculus caudatus* (Fig. 2). In comparison to the genera *Rocahepevirus*, *Avihepevirus* and *Chirohepevirus*, these shrew HEVs share relatively close phylogenetic relationships to *Palsahepevirus*, which includes the prototype human HEV, though they remain divergent from either of the two *Paslahepevirus* species. The topology of the phylogenetic tree of full-length genome sequences was generally consistent with that of ORF1 (Fig. S1). Our findings expand knowledge about the worldwide prevalence and genetic diversity of orthohepeviruses in small mammals.

According to ICTV, the taxonomy of hepeviruses species is based on the phylogenetic analysis of partial amino acid sequence from the methyltransferase, RNA-directed RNA polymerase and the capsid proteins (https://ictv.global/report/chapter/hepeviridae/hepeviridae). However, the species or genus demarcation criteria are not specified, and many newly identified HEV variants have not been taxonomically assigned. Due to their phylogenetic divergence and low similarity to other hepeviruses, the shrew HEV Co-KY2016, along with the other HEV variants from *Crocidura* spp. shrews, is likely to be assigned at least as an independent species within *Palsahepevirus*. However, whether they represent a new species or even a new genus needs to be determined through further analysis.

## Discussion

Here, we identified the full-length genome of two novel rodent and shrew orthohepeviruses from Kenya, compared their genome sequences, and analysed their phylogenetic relationship with other hepeviruses. Previous studies revealed a strong correlation between the phylogeny of hepeviruses and that of corresponding host taxa, suggesting the long-term coevolutionary relationship between the viruses with their hosts [12]. In our analysis, clustering of hepeviruses from the same or similar *Crocidura* shrew species regardless of geographical distance [12,17, 18], as well as the distinct separation between the shrew HEVs and other hepeviruses, may provide more evidence for the virus-host co-speciation in the evolutionary history of hepeviruses.

Rodents have been recognized as important reservoirs of hepeviruses, as they are the major hosts of the genus *Rocahepevirus* (HEV-C) and harbour a high diversity of HEV-C strains [19]. Previous studies reported that rodents may also be infected with human HEV, further suggesting the possible cross-species transmission of hepeviruses among humans and rodents [20]. Moreover, an evolution study demonstrated that rodents may act as major drivers of hepevirus genealogy, and the ancient origin of paslahepeviruses may be even associated with rodent hepeviruses [17]. Besides rodents, we and other groups have discovered diverse hepeviruses in different species of shrews [12,17, 18]. These findings suggest that shrews may be a previously neglected reservoir for hepeviruses. Uncovering and sequencing more novel hepeviruses from shrews in the future will help elucidate the role of these small mammals in hepevirus evolution.

Rodents and shrews are two large groups of small mammals known as zoonotic sources of emerging viruses such as hantaviruses, parahenipaviruses and Borna disease virus [21,22]. Acute hepatitis in humans caused by the interspecies transmission of animal paslahepeviruses, especially swine HEV, has been documented for a long time. In recent years, individual teams from Spain, Canada, France and China reported events of human infection by rat-derived rocahepeviruses that are distinct from prototypical human and swine HEV, which has drawn increasing attention [23,26]. Importantly, previous infection with conventional human HEV fails to provide cross-protection against rat HEV infection due to their high divergence in the antigenic properties, enhancing the risk of rat HEV to public health [27]. In addition to humans, rat HEV may also jump to other animal hosts. Previous studies have provided experimental evidence affirming that rat HEV is capable of infecting non-human primates and pigs [28,29]. Epidemiological survey also revealed herd prevalence of rat HEV infection in pig farms in Spain [30]. These findings suggested that other animals may serve as the vectors or intermediate hosts for rat HEV amplification and transmission, further increasing the likelihood of rat HEV emergence in humans.

Though we did not have experimental or epidemiological evidence for the spillover of rat HEV Rr-KY2016, its close similarity to another rat HEV identified in a human patient, with their capsid proteins (ORF2) sharing 94.7% amino acid sequence identity, implies its possible zoonotic capacity (Table S1) [26]. Moreover, a similar hepevirus strain was detected in the same rat species from Sierra Leone, indicating a wide circulation of related rat HEV in Africa. These rat HEV may have the potential to cause sporadic disease outbreaks among local human populations in Africa. Regarding the shrew HEV, little information is available about the currently known strains beyond their genome sequences, no matter of Co-KY2016 or those from Ethiopia or Germany. Future studies that analyse their virological features and investigate their spillover potential are, therefore, needed. Considering a relatively high proportion of HIV-infecting immunocompromised populations plus the inadequate sanitation and poor hygiene level in some areas, which can facilitate the virus spread, the threat that zoonotic hepeviruses pose to human health in Africa should not be underestimated. Proactive surveillance of hepeviruses targeting rodents and shrews as wildlife reservoirs is thus warranted for early identification of novel hepevirus strains with cross-species transmission risk.

In conclusion, the current study is the first to report and analyse the full-genome sequences of orthohepeviruses from rats and shrews in Kenya. The newly sequenced rat HEV is closely related to other rat-derived HEV-C strains, including ones that are known to have caused zoonotic infection, while the novel shrew HEV may be putatively classified to a novel *Paslahepevirus* species or a novel genus in the subfamily *Orthohepevirinae*. This study suggests the spillover potential of hepeviruses carried by rats and shrews in Kenya. It provides a basis for further functional studies on these viruses, which can help improve understanding of their cross-species infection risk and enhance preparedness against future disease emergence.

## Supplementary material

10.1099/mgen.0.001538 Uncited Supplementary Material 1.

## Abbreviations

HEV: hepatitis E virus
ICTV: International Commitee on Taxonomy of Viruses
NCBI: National Center for Biotechnology Information
NGS: next-generation sequencing
