# Supplementary material for: Genomic characterization of novel orthohepeviruses in shrews and rats from Kenya
Source: Microb Genom. 2025 Dec 5;11(12):001538. doi: 10.1099/mgen.0.001538 (PMC12680325; doi:10.1099/mgen.0.001538)
Supplement: Uncited Supplementary Material 1. [file mgen-11-01538-s001.pdf]

Supplementary Materials for

Genomic characterization of novel orthohepeviruses in shrews and rats from Kenya

Carol Vannesa Nawenja[1,2#], Griphin Ochieng Ochola[1,3#], Vincent Obanda[4], Sheila Ommeh[5], Xing-Lou Yang[6], Yan Zhu[1], Bei Li[1], Jie-Wen Deng[1,2], Bernard Agwanda[3]*, Ben Hu[1]*

1. State Key Laboratory of Virology and Biosafety, Wuhan Institute of Virology, Chinese Academy of Sciences, Wuhan, People's Republic of China
2. University of Chinese Academy of Sciences, Beijing, People's Republic of China
3. Department of Mammalogy, National Museums of Kenya, Nairobi, Kenya
4. Veterinary Science and Laboratories Department, Wildlife Research and Training Institute, Naivasha, Kenya
5. Center for Animal Science, Queensland Alliance for Agriculture & Food Innovation, the University of Queensland, Brisbane, Queensland, Australia
6. Key Laboratory of Genetic Evolution & Animal Models, Kunming Institute of Zoology, Chinese Academy of Sciences, Kunming, China

# These authors contributed equally to this work as first authors.
Corresponding author
Dr. Ben Hu, Mr. Bernard Agwanda
huben@wh.iov.cn, benrisky@gmail.com

Supplementary Tables

Table S1. Comparison of the full-length genome, ORF1 and ORF2 sequence identity percentage between rat HEV Rr-KY2016 and representative HEV strains within the *Rocahepevirus* genus from various host species

Strain name	GenBank accession No.	Host species	Virus species	Sampling location	Sequence identity (%)				
					Full-length genome	ORF1		ORF2	
						nucleotide	amino acid	nucleotide	amino acid
22072190255_HEV-C	OP610066	*Homo sapiens*	*Rocahepevirus ratti*	France	86.9	85.9	95.0	89.2	94.7
Ra. rattus_hepe_1	PQ541186	*Rattus rattus*	*Rocahepevirus ratti*	Sierra Leone	84.9	83.0	92.8	87.5	96.4
LA-B350	KM516906	*Rattus norvegicus*	*Rocahepevirus ratti*	USA	83.7	82.9	92.6	85.8	96.1
rat/R63/DEU/2009	GU345042	*Rattus norvegicus*	*Rocahepevirus ratti* (Genotype C1)	Germany	83.6	82.6	91.8	86.1	95.7
ratELOMB-131	LC145325	*Rattus rattus*	*Rocahepevirus ratti*	Indonesia	77.7	76.7	88.0	79.7	92.4
FRHEV4	JN998606	*Mustela putorius*	*Rocahepevirus ratti* (Genotype C2)	Netherlands	69.6	68.5	75.6	72.0	79.4
Ac14/LiJiang/2015	MG020022	*Apodemus chevrieri*	*Rocahepevirus ratti* (Genotype C3)	China	66.1	64.5	68.2	69.4	74.9
Em40/LuXi/2014	MG020024	*Eothenomys melanogaster*	*Rocahepevirus eothenomi*	China	58.2	56.3	56.9	60.8	63.6
KS_10_2645/GER/2010	MK192406	*Microtus arvalis*	*Rocahepevirus eothenomi*	Germany	58.3	57.4	55.5	60.5	63.4
RtCb-HEV/HeB2014	KY432899	*Cricetulus barabensis*	unassigned species	China	53.9	52.0	52.9	59.2	60.0
RtCm-HEV/XJ2016	KY432903	*Cricetulus migratorious*	unassigned species	China	58.0	54.8	56.3	61.3	63.3

Table S2. Sequence information of primers used in gap filling of the full-length genome sequencing of the rat HEV Rr-KY2016 and shrew HEV Co-KY2016.

Gap filling primer sequences (5'-3')		
Rr-KY2016 Primer Set 1	Round 1	Forward: TATGGAGTTGAAGGAGTACACCTG
		Reverse: CATAATGTGTGTGTTAGACCCATC
	Nested PCR	Forward: CACTCTGGTCTGTAGCATGTGTG
		Reverse: GTTAAAGGTGACGTGGAAAGGTTG
Rr-KY2016 Primer Set 2	Round 1	Forward: ATGGCAAGGTATTACATAAAGAGAC
		Reverse: ATAACTAGACACTATCGGCTGCTG
	Nested PCR	Forward: CTGGCAGCAGGGAACAACTAAG
		Reverse: AGGTGGAAAGCCCGGCCATAG
Co-KY2016 Primer Set	Round 1	Forward: ATTCATTCAACTGAGCTCTTGC
		Reverse: TGCACAGTGTCACTCAGCCTAAG
	Nested PCR	Forward: ATCGGCTGCCACTTTGTGATTC
		Reverse: GTAAGATGGCTGCAACCTCTTG

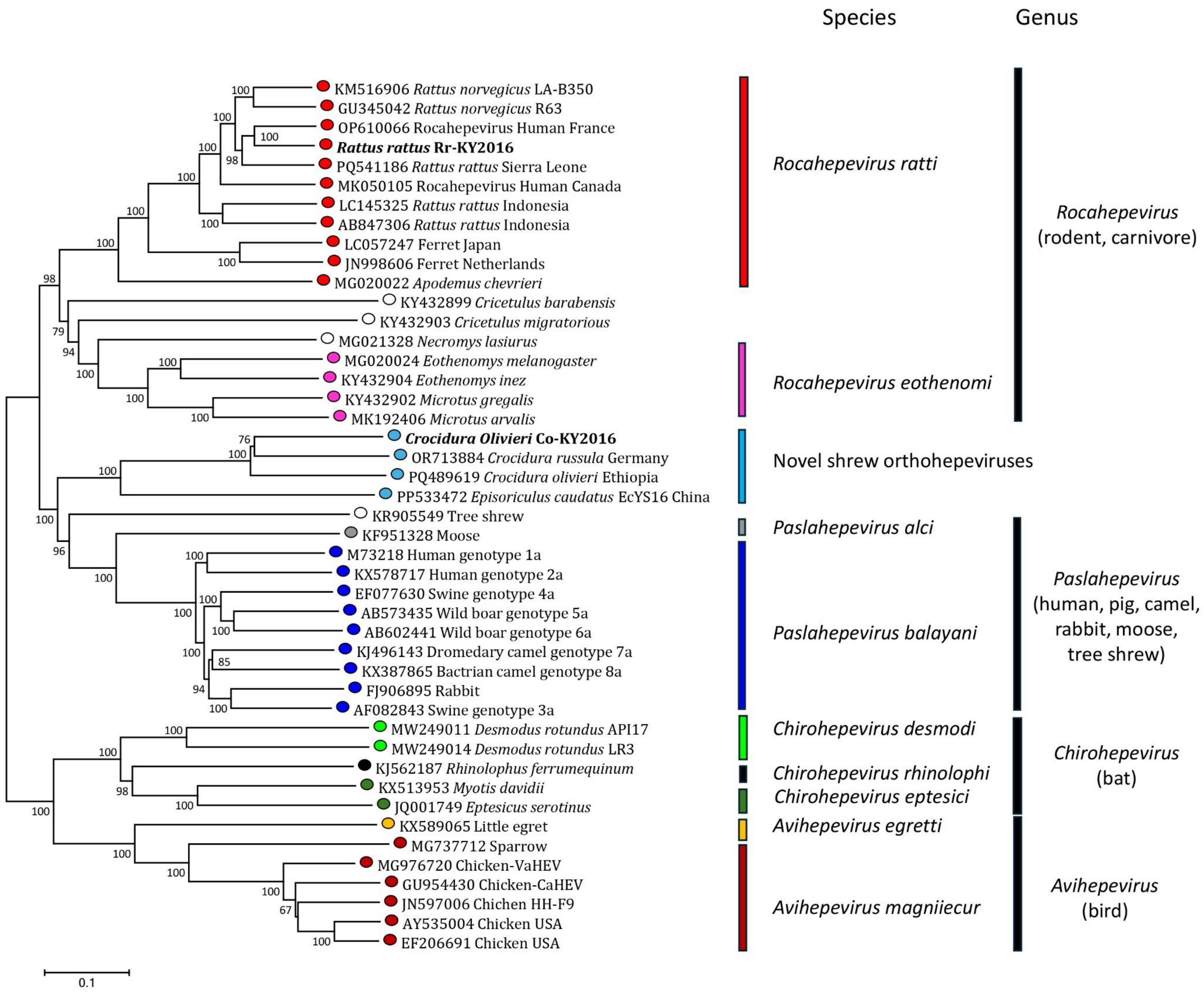

Fig S1. Phylogenetic trees of the full-length genome sequences of the novel shrew HEV, rat HEV and related orthohepeviruses.

The neighbor-joining tree was constructed using Jukes-Cantor model with pairwise deletion, based on full-length genome sequences of shrew HEV Co-KY2016, rat HEV Rr-KY2016 and 43 representative members within the family *Hepeviridae*. Viruses belonging to different species assigned by ICTV are represented by circles in different colors. The host species information of all listed hepeviruses from rodents and shrews are shown. The newly sequenced shrew and rat HEVs are highlighted in bold. Scale bars, nucleotide substitutions per site.
